# Exploring activity compensation amongst youth and adults: a systematic review

**DOI:** 10.1186/s12966-022-01264-6

**Published:** 2022-03-12

**Authors:** Brittany A. Swelam, Simone J. J. M. Verswijveren, Jo Salmon, Lauren Arundell, Nicola D. Ridgers

**Affiliations:** Institute for Physical Activity and Nutrition, School of Exercise and Nutrition Sciences, Deakin University, 221 Burwood Highway, Burwood, Geelong, VIC 3125 Australia

**Keywords:** Activity compensation, Activitystat, Physical activity, Sedentary behaviour, Youth, Adults

## Abstract

**Background:**

Globally, significant efforts have focused on increasing physical activity and reducing sedentary behaviour in youth and adults across a range of settings (e.g., schools, workplaces, community, and home). Despite this, interventions have had varied efficacy and typically have failed to sustain changes in behaviours over time. One explanation that has been put forth to explain the mixed success of interventions is activity compensation. However, little is known about activity compensation, including whether compensation occurs, and perceptions and potential mechanisms of activity compensation. Understanding activity compensation would assist in tailoring and targeting of potential intervention strategies. The primary aim of this review was to synthesise research that has investigated activity compensation in youth and adults. The secondary aim was to identify potential reasons for and/or awareness of compensatory changes that may have occurred.

**Methods:**

An electronic search of the EBSCOhost (via Academic Search Complete, CINAHL Complete, Education Source, Health Source: Nursing/Academic Edition, PsycINFO, SPORTdiscus with Full Text), MEDLINE Complete, Global Health, EMBASE, Scopus and Web of Science databases up to May 2021 was conducted. Quality assessment of included quantitative studies used a modified compensation-specific McMaster Quality Assessment Tool.

**Results:**

A total of 44 studies met the inclusion criteria (22 = adult populations; 22 = youth populations) and were classified as (1) quantitative (*n* = 31); (2) combination of quantitative and behavioural (*n* = 11); (3) behavioural only (*n* = 1); and (4) qualitative (*n* = 1). Of the 42 studies that included a quantitative component, 11 (26%) reported compensation occurred. Within the 13 studies examining specific behaviours, 35 behaviours were assessed, and evidence of compensation was inconsistent. Compensation mechanisms included fatigue, time constraints, lack of motivation, drive to be inactive, fear of overexertion, and autonomous motivation.

**Conclusion:**

Little evidence of compensation was reported in the included quantitative studies; however, inconsistencies between studies makes comparisons difficult. There was considerable variability in the types of behaviours assessed in quantitative studies, and few studies examined potential compensatory mechanisms. Future research, using compensation specific study designs, methods, and analytic techniques, within different population sub-groups, should address these evidence gaps.

**Supplementary Information:**

The online version contains supplementary material available at 10.1186/s12966-022-01264-6.

## Introduction

Regular engagement in physical activity confers physical and mental health benefits in both youth (5–18 years old) and adult populations, including favourable cardiometabolic biomarkers, improved cognition and well-being [1, 2], and among adults, lower risk of all-cause mortality [3, 4]. Conversely, higher levels of sedentary behaviours such as screen time are associated with negative physical and mental health outcomes in youth [5], as well as cardiometabolic diseases, cancer incidence, and depression in adults [6, 7]. Globally, 75% of countries participating in the Active Healthy Kids Global Alliance on physical activity for children and youth (*n* = 49) reported that over 80% of children did not meet the daily moderate- to vigorous-intensity physical activity (MVPA) guidelines of 60 min per day [8]. Moreover, a pooled analysis of 1.6 million adolescents and of 1.9 million adults found 81% [9] and 28% [10], respectively, failed to meet their specific physical activity guidelines [11]. Significant efforts have focused on increasing physical activity and reducing sedentary behaviour across all age groups and in a range of settings (e.g., schools, work places, community, and home) [12–15], yet interventions have had varied efficacy and have typically failed to sustain changes in behaviours over time [13, 16–18].

One potential explanation for such varied intervention efficacy is activity compensation. It has been hypothesised that activity levels may be under some degree of biological control (an ‘activitystat’), which operates in the same way as the homeostatic mechanisms that regulate body temperature, blood pH, and fluid balance within the body [19]. Specifically, the activitystat hypothesis posits that physical activity levels are kept within tolerable activity levels or energy expenditure ranges (activity set-points), meaning that intensity, frequency, duration and/or load of activity may increase or decrease in response to a perturbation (e.g., an activity intervention) to compensate for the additional (or lack thereof) activity [20]. It is crucial to highlight the importance of such changes occurring in response to a perturbation, as this is what sets compensatory responses apart from habitual activity. In addition, as all activity intensities would contribute to the total activity set-point, the compensatory responses would be expected to occur across the activity spectrum (i.e. sedentary behaviour [SED], light physical activity [LPA], and MVPA) [21]. Upon removal of the perturbation, activity levels are hypothesised to return their original levels [22]. This may explain why interventions have limited efficacy for sustained change in activity levels. Despite this, past behavioural activity research has mostly focused on the impact of social and environmental variables on behaviours, largely neglecting the potential biological basis for activity [19, 23].

In a 2013 review of studies examining activity compensation, Gomersall and colleagues [24] reported that 63% (5/8) of child studies, 40% (6/15) of adult studies and 80% (4/5) of elderly studies indicated compensation had occurred [24]. Whilst Gomersall and colleagues [24] focused on experimental and intervention studies, which enables changes in activity levels to be examined under controlled conditions [24], observational studies that can provide insights into individual day-to-day variability in activity were excluded [25]. Further, though compensation is hypothesised to be a biological response, the way in which any responses are observed or potential reasons for occurring has not been reviewed to date. Specifically, it is unknown what behaviours may change and the potential mechanisms underlying such changes. Consequently, there is a need to synthesise activity compensation evidence with methodological considerations and examine any potential reasons as to why compensation may occur (if at all).

The primary aim of this systematic review was to synthesise research that has investigated activity compensation in youth and adults. The secondary aim was to identify and examine any reasons for and/or awareness of compensatory changes that may have occurred.

## Methods

### Protocol and registration

The systematic review was registered with PROSPERO (CRD42019133914). The review was conducted in accordance with the Preferred Reporting Items for Systematic Reviews and Meta-Analyses (PRISMA) guidelines [26]. The PRISMA Checklist is provided in Supplementary Information 1.

### Search strategy

An electronic search of the EBSCOhost (via Academic Search Complete, CINAHL Complete, Education Source, Health Source: Nursing/Academic Edition, PsycINFO, SPORTdiscus with Full Text), MEDLINE Complete, Global Health, EMBASE, Scopus and Web of Science databases up to May 2021 was conducted. The search strategy was developed in conjunction with a research librarian with key words in the following areas: activity compensation ([compensation and physical activity or sedentary or exercise or energy expenditure or energy balance] or [ActivityStat or EnergyStat or energy displacement]) and age ([child or youth or adolescent] or [adult]). The full search strategy, including proximity search strategy functions and truncations, for the different databases can be found in Supplementary Information 2. All titles and abstracts were screened in full and independently by two reviewers (B.S., and S.V. or N.R) using the Cochrane review production platform Covidence (Veritas Health Innovation; Melbourne, Australia). Discrepancies were recorded through Covidence and reviewed by three researchers (B.S., S.V. and N.R.) until a consensus was reached. In the case that a consensus could not be reached, discrepancies were discussed with the research team. Agreement between reviewers in the title/abstract stage was 91%. Full text articles that met the initial screening criteria were then independently screened for eligibility to be included in the review by two researchers (B.S. and S.V.), and inconsistencies were again discussed and resolved with the research team where required. Agreement between reviewers was 72%. The reference lists of studies deemed eligible for inclusion were searched for additional relevant studies for potential inclusion [27].

### Eligibility criteria

All original study designs were considered for inclusion. Studies were eligible if they met the following criteria: (a) participant’s mean age was 5–65 years; (b) focused on the general population, i.e., the target population did not solely focus on participants with chronic conditions, athletes, or overweight/obesity (as they may have different compensation ‘drivers’ such as chronic pain, muscular atrophy, etc.); (c) the study explicitly undertook analyses designed to examine activity compensation or compensatory responses, or explored compensatory responses as part of their methods (i.e., study was designed to examine changes in activity across the activity spectrum, or between settings, and used compensation when describing their results); (d) was published in English; and (e) was published between January 1999 to May 2021. The start date was selected to align with the first publication outlining the activitystat hypothesis (1999) [19]. Quantitative studies that, for example, were not designed to look at similarities or differences in activity between settings or time periods, but rather used compensation as a discussion point were not included. Quantitative and qualitative studies were included if they discussed potential mechanisms, reasons, or insights into activity compensation. Articles that were published ahead of print and had a DOI were also eligible for inclusion. Abstracts, conferences, reviews, study protocols, and dissertations were not eligible for inclusion.

### Data extraction

For this review, studies were classified into four categories: 1) Quantitative only (i.e. measuring compensation quantitatively); 2) Quantitative and behavioural (i.e. quantitative compensation studies that also recorded behaviours, this included studies measuring mechanisms/perceptions of compensation); 3) Behavioural only (i.e. a non-qualitative assessment of behaviours, perceptions of compensation, or mechanisms); and 4) Qualitative only. This approach was used to distinguish between studies that were eligible for inclusion in this review but examined different aspects of activity compensation. Quantitative data were extracted by one reviewer (B.S). For consistency purposes, 15% of articles were extracted and reviewed by another reviewer (S.V.). Data were extracted using a standardised form and included: study/participant characteristics (e.g. mean age, study design, % male/female, % overweight/obesity, etc.), outcomes examined (e.g. sedentary time), activity assessment method (e.g. pedometer, accelerometer), study design (e.g. cross-sectional), activity compensation methodological considerations (e.g. timeframe examined, analytical approach), reported results (e.g. compensation reported), and behavioural assessments (if any; e.g. sitting time in different locations, active transport, etc.). The authors then reviewed the information extracted and clarified where any differences in information were identified. Support was provided via discussion with the remaining authors if clarifications were required (e.g., what analytical approaches were used). The remaining data were re-checked and verified by one reviewer (B.S.). Qualitative data were extracted by one reviewer using thematic synthesis (B.S.) [28].

### Quality assessment

A quality assessment tool, derived from the McMaster Quality Assessment Tool [29] and compensation specific criteria as defined by Rowlands [30], was developed by the research team. The tool was used to assess the included quantitative studies only (categories 1, 2, and 3). Nine compensation specific criteria were developed [30] and included: study design (i.e. experimental design as the ‘gold standard’), implementing activity during inactive times and/or restricting activity during inactive times (i.e. when perturbation occurred), measuring activity across settings, sensitivity of measurement tools, analytical approach (e.g. within-group), and assessed the whole activity spectrum (i.e. SED to MVPA) [21]. In total, 16 criteria, including general and compensation-specific items, were used to assess quality across six overarching categories of (a) selection bias (e.g. is the sample representative); (b) study design; (c) data collection (e.g. is the measurement tool objective, valid and reliable); (d) withdrawals and dropouts (e.g. % of dropouts reported); (e) exposure integrity (e.g. % of participants receiving allocated exposure/intervention); and (f) analyses (e.g. within/between-person analyses). The compensation specific criteria were included across all categories except the withdrawals/dropouts. For category 1, 2, and 3 studies, a quality rating of strong, moderate, or weak was given to each component, except for dichotomous variables that were rated strong or weak. In the event a component could not be clearly determined from the paper, a weak rating was given. No overall study quality score was given in line with current recommendations [31]. Category 4 papers were assessed using the McMaster Qualitative Review Form [32]. The category 4 paper was not given a rating according to the review form guidelines [32]. The full quality assessment tools can be found in the Supplementary Information 3 and 4.

## Results

### Description of included studies

**Fig. 1:**
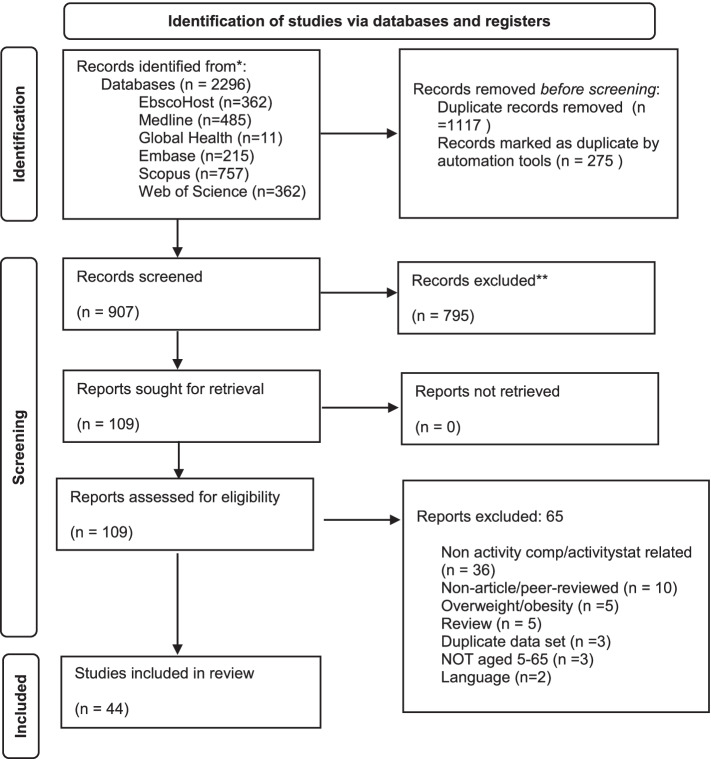
PRISMA Flow Diagram. 2020 PRISMA flow diagram [26] of studies assessed for eligibility and included in review

Extracted data were analysed between May 2021–July 2021. Of the studies initially identified, 109 full-text studies were screened, and 44 studies were included in the review. Of these, 31 were classified as quantitative only (category 1) [20, 21, 33–61], 11 assessed quantitative outcomes but included subjective behavioural components (category 2) [62–72], one examined self-reported behaviours only (category 3) [73], and one qualitative study examined mechanisms and perceptions of compensation (category 4) [74]. The PRISMA flowchart can be found in Fig. 1.

The characteristics of the included studies are found in Table 1. Studies were conducted in 10 different countries (see Table 1), with the majority occurring in the USA (*n* = 13), the UK (*n* = 13) and Australia (*n* = 9). The age of participants ranged between 5 [72] to 63 [67] years, with 50% studies specifically focusing on children and/or adolescents (*n* = 22; [20, 21, 33, 36, 39, 41–44, 47–52, 55, 58, 63–65, 71, 72]) and 50% focusing on adults (*n* = 22; [34, 35, 37, 38, 40, 45, 46, 53, 54, 56, 57, 59–62, 66–70, 73, 74]). Study sample sizes ranged from 16 participants [35, 45] to 12,969 [69] participants. Of the 44 studies included, the primary or secondary aim of 30 [20, 21, 33, 35–39, 41–44, 48, 50–56, 59, 64, 66, 68–74] and eight studies [34, 38, 40, 47, 57, 58, 62, 63], respectively, was to examine activity compensation. The remaining six were ‘unspecified’ (e.g., results included compensation analyses but this was not a specified aim) [45, 46, 49, 60, 61, 67]. Studies were primarily cross-sectional (52%), followed by experimental (randomised crossover *n* = 7; randomised experiment *n* = 2; pre-post *n* = 1; two-phase single case *n* = 1) (25%), and randomised controlled trials or intervention studies (18%). There was one longitudinal [69] and one qualitative study [74] included in the review.

**Table 1:** Description of reviewed studies

Author(s)/Study date	Country	Study Design	Age group	Compensatory Aim	Study Classification	Population Characteristics
Bagget et al. [20]	USA	Cross-sectional	Youth	Primary	Quantitative	Sample*:* 3440 girls (2005), 3467 girls (2006) (6916 girls total) Mean age*:*14 years
Carlson et al. [33]	USA	Cross-sectional	Youth	Primary	Quantitative	Sample: 528 adolescents Mean age*:* 14.12 years
Clemes et al. [62]	UK	Cross-sectional	Adult	Secondary	Quantitative/Behavioural	Sample: 72 full-time office workers Mean age: 37 years
Clemes et al. [34]	UK	Cross-sectional	Adult	Secondary	Quantitative	Sample: 170 office workers Mean age: 40.1 years
Costigan et al. [63]	Australia	RCT	Youth	Secondary	Quantitative/Behavioural	Sample: 65 adolescents, 3 PE lessons, grades 9–10 (1 secondary school) Mean age: 15.8 years
Cull et al. [35]	USA	RCT	Adult	Primary	Quantitative	Sample: 16 healthy adults who met recommended PA guidelines weekly ≥150 MVPA per week, 2 cohorts (*n* = 8 and *n* = 8) Mean age: *Control:* 21.6 years *Intervention:* 22.4 years
Dale et al. [36]	USA	Experimental (crossover)	Youth	Primary	Quantitative	Sample: 78 children, Years 3–4, private elementary school Mean age: 9.3 years
DiBlasio et al. [37]	Italy	Experimental (pre-post)	Adult	Primary	Quantitative	Sample: 41 postmenopausal women enrolled in study Mean age: 55.9 years
Fremeaux et al. [64]	UK	Cross-sectional	Youth	Primary	Quantitative/Behavioural	Sample: 215 children, aged 8–10; 3 primary schools Mean age: Not reported
Gomersall et al. [38]	Australia	RCT	Adult	Primary	Quantitative	Sample: 129 previously inactive adults Sample size by group: *Control:* (*n* = 43) *Moderate:* (*n* = 43) *Extensive:* (*n* = 43) Mean age: 41 years
Goodman et al. [65]	UK	Cross-sectional	Youth	Secondary	Quantitative/Behavioural	Sample*:* Combo of 2 samples, 11 schools, 345 children Sample 1: 194 children Years 6–8 (aged 10–13) Sample 2*:* 151 children Years 4–6 (aged 8–11)
Gray et al. [74]	UK	Qualitative	Adult	Primary	Qualitative (interviews)	Sample: 9 of 14 ‘compensators’ identified from previous study Mean age: 58.56 years
Jakubec et al. [39]	Czech Republic	Cross-sectional (time series)	Youth	Primary	Quantitative	Sample: 2702 students, 959 full inclusion Mean age: *Boys:* 16.6 years *Girls:* 16.5 years
Jans et al. [66]	Netherlands	Cross-sectional	Adult	Primary	Quantitative/Behavioural	Sample: 7724 Dutch workers Mean age: 39 years
Liguori et al. [40]	USA	Cross-sectional	Adult	Secondary	Quantitative	Sample: 84 college students (33 cadets, 51 non cadets) Mean age*:* 20.26 years
Long et al. [41]	USA	Cross-sectional	Youth	Primary	Quantitative	Sample: 2548 participants Mean age of participants: *boys aged 6–11*: 9.1 years *boys aged 12–19*: 14.7 years *girls aged 6–11:* 9.2 years *girls aged 12–19:* 15.0 years
Mackintosh et al. [42]	UK	Cross-sectional	Youth	Primary	Quantitative	Sample*:* 25 healthy age and sex matched controls Mean age: 11.7 years
Massie et al. [43]	UK	Controlled trial (non-randomised)	Youth	Primary	Quantitative	Sample: 31 girls from 2 secondary schools *exercise*: 15 girls *control*: 16 girls Mean age: not reported, 12–15 years
Matthews-Ewald et al. [44]	USA	Cross-sectional	Youth	Primary	Quantitative	Sample: 268 9th and 10th grade students Mean age: not reported
Matthews et al. [67]	USA	Cross-sectional	Adult	Unspecified	Quantitative/Behavioural	Sample: 1020 adults Mean age: 63.1 years
McCormack et al. [68]	Australia	Cross-sectional	Adult	Primary	Quantitative/Behavioural	Sample: 1803 adults, top tier and bottom tier of social advantage Mean age: not reported, 26% 18–29, 29% 30–39, 27% 40–49, 18% 50–59
McLaughlin et al. [45]	UK	Experimental (crossover)	Adult	Unspecified	Quantitative	Sample: 16 adults Mean age: *Males:* 23 years *Females:* 24 years
Meijer et al. [46]	Netherlands	Controlled trial (non- randomised)	Adult	Unspecified	Quantitative	Sample: 22 participants (15 exercise group, 7 control group) Mean age by group: *Exercise:* 58.9 years *Control:* 57.4 years
Morgan et al. [47]	USA	Cross-sectional (time-series)	Youth	Secondary	Quantitative	Sample: 485 6th graders Mean age: not reported
Nooijen et al. [69]	Sweden	Longitudinal	Adult	Primary	Quantitative/Behavioural	Sample: 12,969 adults *Mean age:* 45 years
O’Sullivan et al. [48]	USA	Experimental (randomised crossover)	Youth	Primary	Quantitative	Sample: 33 children Mean age: 8.7 years
Penning et al. [49]	Australia	Experimental (randomised crossover)	Youth	Unspecified	Quantitative	Sample: 18 adolescents Mean age: 13.5 years
Radtke et al. [73]	Switzerland	Cross-sectional	Adult	Primary^a^	Behavioural	Population: 135 adults Mean age: 32.23 years
Ridgers et al. [50]	Australia	Cross-sectional	Youth	Primary	Quantitative	Sample: 127 children Mean age by group: *ActiGraph sample:* 10.4 years *Sensewear sample:* 10.5 years
Ridgers et al. [51]	Australia	Experimental (randomised experiment)	Youth	Primary	Quantitative	Sample: 158 children (accelerometry, survey, and log book), 149/158 to wear additional SenseWear Mean age: 11.3 years
Ridgers et al. [52]	Australia	Cross-sectional (time-series)	Youth	Primary	Quantitative	Sample: 235 children, 9 primary schools, Years 4–5, from PHASE study Mean age: 10.1 years
Ridgers et al. [21]	Australia	Cross-sectional	Youth	Primary	Quantitative	Sample: 248 children, 9 primary schools, Years 4–5, PHASE study Mean age: 10 years
Rocha et al. [54]	UK	Experimental (randomised crossover)	Adult	Primary	Quantitative	Sample: 20 adults Mean age by group: *Active group:* 22.6 years *Inactive group:* 22.3 years
Rocha et al. [53]	UK	Experimental (randomised crossover)	Adult	Primary	Quantitative	Sample: 30 participants Mean age by group: *Active group:* 22.5 years *Inactive group:* 23.8 years
Saunders et al. [55]	Canada	Experimental (randomised crossover)	Youth	Primary	Quantitative	Sample: 20 healthy children and youth Mean age: *Males:* 12.8 years *Female:* 11.3 years
Schubert et al. [56]	USA	Intervention	Adult	Primary	Quantitative	Sample: 24 adults Mean age: 29.5 years
Schutz et al. [57]	Switzerland	Experimental (randomised experiment)	Adult	Secondary	Quantitative	Sample: 55 normal-weight and overweight women Mean age: 27 years
Siddique et al. [70]	USA	RCT	Adult	Primary	Quantitative/Behavioural	Sample: 204 adults Mean age: 33 years
Stylianou et al. [58]	USA	Experimental (crossover)	Youth	Secondary	Quantitative	Sample: 49 primary school children, Years 3–4, 2 schools Mean age: not reported
Tanaka et al. [71]	Japan	Cross-sectional	Youth	Primary	Quantitative/Behavioural	Sample: 426 primary school children Mean age: 9.3 years
Tigbe et al. [58]	UK	Cross-sectional	Adult	Primary	Quantitative	Sample: 112 participants Mean age by group: *Delivery staff:* 38 years *Office staff:* 40 years
Turner et al. [60]	UK	RCT	Adult	Unspecified	Quantitative	Sample: 41 participants Mean age: 54 years
Vandelanotte et al. [61]	Australia	Cross-sectional	Adult	Unspecified	Quantitative	Sample: 1194 shift workers Mean age: 45.3 years
Wilkin et al. [72]	UK	Cross-sectional	Youth	Primary	Quantitative/Behavioural	Sample: *Group 1:* 307 school children (from 53 primary schools) *Group 2:* 215 ‘older’ children from 3 schools *Group 3:* 72 children randomly selected from Glasgow Mean age by group: *Group 1:* tested at 4.9 years & 5.9 years *Group 2:* 9 years old *Group 3:* 5.8 years old

*Abbreviations*: *RCT* Randomised control trial, *PE* Physical education, *PA* Physical activity, *PHASE* Patterns of Habitual Activity Across Seasons Study

^a^Aim examining compensatory health behaviours and physical (in)activity

### Quantitative study overview (categories 1 & 2)

Of the 31 quantitative studies and 11 quantitative/behavioural studies, 11 studies reported evidence of compensation [21, 37, 46, 50, 52, 56, 57, 64, 69, 71, 72], 29 studies reported no evidence of compensation [20, 34–36, 38–45, 47–49, 51, 53–55, 58–63, 65, 66, 68, 70] and two studies had mixed [33] or unclear results [67] (see Table 2).

**Table 2:** Quantitative study description

Author(s)/Study date	Assessment/Data Collection Instruments	Outcomes (PA variables)	Confounders	Timeframe	Analytical Approach	Compensation Reported
Bagget et al. [20]	ActiGraph: hip, waking hours, Treuth et al. cutpoints Data collected 6 consecutive days	LPA, MVPA, total PA: MET-weighted mins, absolute mins, and % of monitored time Inactivity: mins, and % of monitored time spent inactive	Race/ethnicity, socioeconomic status, monitored time, day of week, sample, field centre	Within-day/Between-day	Within-person	No
Carlson et al. [33]	ActiGraph 7164; 71,256; GT1M; GT3X: left hip, waking hours, Evenson youth cutpoints, 30s epochs GlobalSat DG-100 GPS tracker: worn concurrently to accelerometer, waking hours, latitude/longitude every 30s Data collected 7 days, derived from TEAN study	MVPA: mins/day across 5 different locations	All models: Daily time in location (participant mean centred) Moderators*:* participant gender, age, race/ethnicity, and BMI percentile, neighbourhood walkability, income, and parent education.	Within-day	Within-person	Location MVPA & overall MVPA*:* No Between location*:* Yes
Clemes et al. [62]	Pedometer: thigh, waking hours Diary: recording daily pedometer steps recorded at start of day, lunch break, end of afternoon and before bed on work day, start and end of day on weekend, and sitting time Data collected 7 consecutive days	Step count: workdays/non-workdays Sitting time: mins/day	N/A	Within-day	Between-group	No
Clemes et al. [34]	ActiGraph GTIM: waist, Atkin (SED) & Freedson cutpoints (LPA/MVPA), 1 min epochs Data collected 7 consecutive days	SED, LPA, MVPA: working/nonworking hours as % of wear time spent, mins/day	N/A	Within-day	Both	No
Costigan et al. [63]	GENEActiv: wrist, Phillips & Esliger cutpoints Questionnaire: Perceptions of compensation, 3 items, non-validated Data collected across 2 weeks (baseline, experimental week)	MPA, VPA: mean mins	Wear time	Within-day	Within-person	No
Cull et al. [35]	ActiCal: wrist, 30s epochs, Actical adult cutpoints Non-wear time log sheet Data collected 7 consecutive days, measurements at baseline, week 4, and week 9, occurred on 3 occasions	SED, LPA, MPA, VPA: mins/day (average across 7 days)	None	Between-weeks	Both	No
Dale et al. [36]	CSA 7164: waist, 1 min epochs Data collected across 14 weeks overall, worn for 4 school days, 2 non-consecutive days of restricted activity, 2 days of ‘active’ (normal, unrestricted)	CPM: active day, restricted day	None	Within-day	Within-person	No
DiBlasio et al. [37]	SenseWear Pro2: right arm Daily PA measured free-living conditions, 3 consecutive days at baseline(T0), 13-week exercise program, time point 2 (T1) measures occurred 2 weeks before the end of the exercise program	TEE: mean values of total daily energy expenditure MET: intensity of daily physical activities PAEE: time/energy spent on PA activity with an intensity > 3 METS	None	Between weeks (baseline to end of intervention, 11 weeks)	Within-person	Yes
Fremeaux et al. [64]	ActiGraph: hip, 1 min epochs Activity diary: daily activities-type, duration and time Data collected 4 occasions across 12 months, 3 consecutive weeks, accelerometers worn 7 consecutive days	MVPA: mins/day TPA: counts/min	Seasonal variability, age, gender	Between schools/seasons	Between-group	Yes
Gomersall et al. [38]	ActiGraph GT3X: waist, 24/h day (except contact sports and water activities), 30s epochs MARCA: 24-h recall of activities in 5 min intervals, assigned MET value Data collected 5 measurement occasions: baseline (week prior to program start), mid intervention (week 3–4), end of intervention (week 6), 3 and 6-month post-intervention (follow-up) (weeks 12 and 24), worn 7 days each measurement	MVPA, Total activity, TDEE, RMR: min/day, MET minutes	None reported	Between weeks (baseline to follow up, 6 months)	Between-group	No
Goodman et al. [65]	ActiGraph RT3: waist, Rowlands cutpoints (MVPA), 1 min epochs National Travel Survey Diary: adapted for children, cross-checked with children and against accelerometer timings Data collected 4 consecutive days	MVPA: time spent Child Behaviours: % of day spent in MVPA in own home, friend’s home, other home, school lessons, PE/games, clubs and tuition, non-home events, passive travel, school active travel, non-school active travel, structured sport, out of home play, other	Between child analyses: gender, age, weight status and income deprivation	Between-day	Both	No
Jakubec et al. [39]	ActiTrainer: hip, chest strap HRM, waking hours, Tremblay et al. cutpoints, 15 s epochs Data collection 3 consecutive school days	LPA, MVPA, VPA: % mins/day	None reported	Within-day	Between-group	No
Jans et al. [66]	Injuries and Physical Activity in the Netherlands Survey 2000–2005 Data from Injuries and Physical Activity in the Netherlands survey 2000–2005, 1/4 of those who participated were given more detailed questions about sitting time and lying time	SED: sitting time mins/day	Family size, age, gender	Within-day	Between-group	No
Liguori et al. [40]	ActiGraph (ActiTrainer): hip, waking hours, Troiano et al. cutpoints, 60s epochs Data collected 5 consecutive days (2 training days, 3 non-training days), across 4 consecutive weeks, participants randomly assigned to 1 of 4 weeks	MPA, VPA, MVPA: mean mins/day (average weekday, weekend day, week)	None reported	Within-day/Between-day	Both	No
Long et al. [41]	ActiGraph 7164: hip, waking hours, Troiano cutpoints, Trost cutpoints Data obtained from 2003 to 2006 NHANES, 7 consecutive days	MVPA, MPA, VPA: 1 min bouts, 8 of 10 mins bouts of 1 min	Age, gender, income, race/ethnicity, fitness level (baseline), BMI, neighbourhood, school characteristics	Within-day	Within-person	No
Mackintosh et al. [42]	ActiGraph GT3X+: hip, waking hours, Freedson youth cut points, 15 s epochs Data collected 7 consecutive days	SED, LPA, MVPA: mins spent in different intensities	Age, sex, condition, measurement day, wear-time, person-level PA, and/or sedentary time	Between-day	Within-person	No
Massie et al. [43]	Actiheart: chest, 15 s epochs COSMED (gas exchange device): 20 min, supine position Data collected 7 consecutive days in weeks 0, 6, 12, and 18 (12-week supervised exercise intervention)	TEE, AEE: energy expenditure	N/A	Between-weeks (1–18 weeks)	Between-group	No
Matthews-Ewald et al. [44]	ActiGraph GTX3E: hip, Evenson et al. cutpoints, 15 s epochs Data collected 1 week, beginning of term during which participants were enrolled in PE, and 1 week at the end of term. *Only data collected at the start of the term was used due to number of participants	SED, NEAT (LPA), MVPA: mins	Odds ratio: gender, BMI, duration of PE class (in minutes), and number of minutes spent in MVPA and NEAT during PE class Random effects: PE teacher	Within-day	Both	No
Matthews et al. [67]	Activities Completed Over Time in 24 Hours Survey (ACT24) Data collected as 6 recalls over 12 months, randomly selected day every other month	SED, LPA, MPA, VPA, MVPA: hours/day	Age, sex, season of year and day of the week	Not clear	Within-person	Unclear
McCormack et al. [68]	Active Australia Survey: frequency, total duration of PA Data collected from the SEID 1 project	LPA, MPA, VPA: minutes per week	Gender, age, education, and SES	Not clear	Within-person	No
McLaughlin et al. [45]	Oxycongamma online gas analysis system: mouthpiece, 60s intervals Polar Vantage HRM: 8 days habitual, 8 days of exercise program Activity diary: 5 min intervals, match activity to EE Data collected 16 days, 8 days of habitual PA and 8 days of exercise program, 1 week washout for males, ~ 4 weeks washout for females	TEE, AEE, SEDEE, SAEE, SEE: energy expenditure (MJ)	N/A	Not clear	Between-group	No
Meijer et al. [46]	Tri-axial accelerometer: (model not reported), 1 min epochs Data collected over 2-week period, baseline, week 6 and week 12	Counts per day: All days, training days, non-training days	N/A	Between weeks	Both	Yes
Morgan et al. [47]	Pedometers: two worn, right/left side of body Data collected over 2 consecutive weeks, each participant 2–4 days of full data	Steps: mean, daily, PE days, non-PE days	BMI, sex, level of activity	Between-day	Between-group	No
Nooijen et al. [69]	PAQ: average PA behaviour and occupational PA Data collected 4 years apart, via survey 2010 and 2014 (follow-up)	LTE: hours/week, increase or decrease Occupational PA: scale ranged from mainly sedentary-heavy physical work	Age, gender, education	Changes across 4 years, within-day	Between-group	Yes
O’Sullivan et al. [48]	ActiGraph GT3X: hip, 10s epochs, Evenson cutpoints Data collected over 7-day period, baseline week, washout week, 4 experimental days at least 4 days apart, after experimental day there were 3 days of post condition	PAEE: physical activity energy expenditure	Not reported	Within-day/Between-day	Within-person	No
Penning et al. [49]	SenseWear: arm Data collected 48 h post lab sessions	EE: energy expenditure (kJ)	Not reported	Between-day (48 h post)	Within-person	No
Ridgers et al. [50]	ActiGraph: mid-thigh, waking hours, 5 s epochs, Trost cutpoints SenseWear: (subsample) arm, 1 min epochs Data collected 8 consecutive days	ActiGraph: SED, LPA, MVPA, min SenseWear: energy expenditure, kcal	Sex, decimal age, measurement day, wear time, person, year level, physical activity and/or sedentary time or energy expenditure (as appropriate)	Between-day	Within-person	Yes
Ridgers et al. [51]	ActiGraph: hip, 5 s epochs, Freedson cutpoints, waking hours SenseWear: left arm, 1 min epochs, waking hours Data collected over 2-week period, worn Mon-Friday in week 1 and 2	SED, LPA, MVPA: mins/day	Wear time, school	Within-day/Between-day	Within-person	No
Ridgers et al. [52]	activPAL: mid-thigh, waking hours, 15 s epochs Data collected over 8 consecutive days	Sitting time, step time, standing time: mins, weekday, weekend	Sex, year level at school, day of measurement, waist circumference, activPAL wear time	Within-day/Between-day	Within-person	Yes
Ridgers et al. [21]	ActiGraph GT3X+: hip, waking hours, 15 s epochs, Freedson cutpoints Data collected over 7 consecutive days	SED, LPA, MVPA: mins/day	Model 1: sex, grade, day of measurement, waist circumference, and wear time Model 2: In addition, avg. person-level PA and/or sedentary time per day Model 3: In addition, temperature, rainfall, humidity, mins of daylight	Between-day	Within-person	Yes
Rocha et al. [54]	Actiheart: waking hours, 15 s epochs Indirect calorimetry Data collected 3 days, 2 occasions, 4 weeks between experimental conditions	% energy compensation (inclusive of EI)	N/A	Within-day/Between-day	Between-group	No
Rocha et al. [53]	Actiheart: waking hours, 15 s epochs Indirect calorimetry Data collected 7 days between experimental conditions	% energy compensation (inclusive of EI)	N/A	Within-day/Between-day	Between-group	No
Saunders et al. [55]	ActiCal: hip, Puyau cutpoints Ultima PF/PFX metabolic cart: REE/Vo2 Peak Data collected seven consecutive days, 4 occasions (baseline and three experimental conditions), 1 week washout between measurements	SED, LPA, MPA, VPA: min, % total wear time	Condition, wear time, age, sex, Tanner stage, BMI, baseline PA and sedentary behaviour	Not clear, separate statistical analyses of 24 h post indicates between-day	Between-group	No
Schubert et al. [56]	NEPA: ActiGraph GTX3+, non-dominant wrist, 7 days VO2max and RMR: incremental test on a cycle ergometer with breath-by-breath gas collection ParvoMedics TrueOne 2400 Data collected 7 days for ActiGraph, intervention sessions 3x week, 4 weeks	Exercise energy compensation (%)	None	Within-day	Both	Yes
Schutz et al. [57]	Lifecorder uniaxial accelerometer: no info provided Data collected 2-weeks baseline, weeks 3–6 daily walking prescriptions, weeks 7,8 baseline ‘after’	AEE: activity energy expenditure Steps/day: baseline, exercise prescription	None	Between-day	Both	Yes
Siddique et al. [70]	ActiGraph 7164: waist, waking hours, Freedson et al. cutpoints,1 min epochs Data collected 2-weeks baseline, weeks 3–6 daily walking prescriptions, weeks 7,8 baseline ‘after’	SED, MVPA: mins/day	Weekend, accelerometer wear time, gender, age	Between-weeks (2 week blocks, baseline, walking prescription, baseline ‘after’)	Both	No
Stylianou et al. [58]	Pedometer (New Lifestyles NL-1000 model): > 3.6 METs, 4 s epochs PA participation log Data collected 2 weeks at baseline, 5 weeks alternating treatment	MVPA: % of program duration, % of daily MVPA, % school day MVPA Steps: % daily step guideline, % school day steps	BMI, sex, controlled for phase, PE, extra recess	Within-day	Within-person	No
Tanaka et al. [71]	Active style Pro HJA-350IT: hip worn, waking hours Questionnaire: subjectively evaluated screen time Data collected 7 days	LPA, MVPA, VPA: ambulatory, non-ambulatory, and total, expressed as mins/day	Gender, age, body weight, and wearing time	Within-day (PA week totals/7)	Within-person	Yes
Tigbe et al. [59]	ActivPAL: thigh, waking hours Data collected 7 days	Free living PA: Time spent sedentary, time spent upright, time standing, time walking, step count	None	Between-day	Between-group	No
Turner et al. [60]	Actiheart monitor: chest, continuous (day and night), 1 min epochs Data collected 1 week baseline, 24-week intervention (data collected week 2, 9 & 18), 2-week post intervention	Low intensity: < 3 METs, time (mins) and energy (kcal) spent below threshold Mod-vigorous intensity: > 3 METs, time (mins) and energy (kcal) spent above threshold PAEE: physical activity energy expenditure	None	Not clear	Between-group	No
Vandelanotte et al. [61]	IPAQ (long): phone administered Workforce Sitting Questionnaire: phone administered Data collected via telephone, ~ 40 mins per interview, November 2011	Total, occupational and leisure sitting time (over the last 7 days)	Gender, age, education, income, BMI, physical activity and all work variables	Not clear	Within-person	No
Wilkin et al. [72]	MTI (formerly CSA) uniaxial accelerometer: waking hours, Metcalf cut points, 1 min epochs Data collected 7 consecutive days (5 school days, 2 weekend days)	Low intensity, medium intensity, high intensity: weekly total	Seasonality, variation between accelerometers, age, body fat	Between-day	Between-group	Yes

*Abbreviations*: *LPA* Light physical activity, *MVPA* Moderate-to vigorous activity, *PA* Physical activity, *MET* Metabolic equivalent of task, *TEAN* Teen Environment and Neighborhood, *BMI* Body mass index, *SED* Sedentary behaviour, *MPA* Moderate physical activity, *VPA* Vigorous physical activity, *CPM* Counts per minute, *TEE* Total energy expenditure, *PAEE* Physical activity energy expenditure, *TPA* Total physical activity, *MARCA* Multimedia Activity Recall for Children and Adults, *TDEE* Total daily energy expenditure, *RMR* Resting metabolic rate, *HRM* Heart rate monitor, *NHANES* National Health and Nutrition Examination Surveys, *AEE* Activity energy expenditure, *NEAT* Non-exercise activity thermogenesis, *SEID* Study of Environmental and Individual Determinants of Physical Activity, *SEDEE* Sedentary energy expenditure, *SAEE* spontaneous activity energy expenditure, *SEE* Sleeping energy expenditure, *PAQ* Physical Activity Questionnaire, *LTE* Leisure time exercise, *EI* Energy intake, *REE* Resting energy expenditure, *NEPA* Non-exercise physical activity, *IPAQ* International Physical Activity Questionnaire

#### Evidence of compensation

Of the 11 studies reporting evidence of compensation, six were in youth [21, 50, 52, 64, 71, 72] and five were in adult [37, 46, 56, 57, 69] populations. The time frame of compensation included within-day (*n* = 4; [33, 52, 56, 71]) to between-day (*n* = 5; [21, 50, 52, 57, 72]), to between-weeks (e.g. baseline to end of intervention) (*n* = 2; [37, 46]) to between-seasons [64]. All studies used accelerometers, except for one longitudinal study in adults, which assessed compensation within-day at two timepoints (4 years apart) and used the Physical Activity Questionnaire (PAQ) [69]. Outcome variables included energy expenditure [37], steps [57], counts per minute [64] and counts per day [46], LPA [71] and MVPA [64, 71] (Table 2). Only two studies, both conducted with youth, examined compensatory changes across the full waking activity spectrum (SED, LPA, MVPA) [21, 50]. Six studies used a within-person design [21, 33, 37, 50, 52, 71], whilst three studies used between group analyses [64, 69, 72]. Three studies used both within-person and between-person or between- group analyses [46, 56, 57]. One study (adolescent population) [33], reported that compensation only occurred ‘between locations’ (Table 2).

#### No evidence of compensation

Of the 29 studies reporting no evidence of compensation, 15 were conducted in youth populations [20, 36, 39, 41–44, 47–49, 51, 55, 58, 63, 65] whilst 14 were conducted in adult populations [34, 35, 38, 40, 45, 53, 54, 59–62, 66, 68, 70]. The time frame examined varied from within-day (*n* = 9; [34, 36, 39, 41, 44, 58, 62, 63, 66]) and (*n* = 6; [20, 40, 48, 51, 53, 54])/or between-day (*n* = 5; [42, 47, 49, 59, 65]) to between-weeks (e.g. pre, mid, and post intervention (*n* = 3; [38, 43, 70]). The majority (90%) of studies used device-based measures of activity, primarily accelerometers (*n* = 23; [20, 34–36, 38–45, 48, 49, 51, 53–55, 59, 60, 63, 65, 70]) and pedometers (*n* = 3; [47, 58, 62]). Two studies subjectively measured adults’ physical activity using surveys [67, 68]. Five studies (three in youth, two in adults) examined the whole activity spectrum [34, 35, 42, 51, 55]. One study, conducted with adolescents, examined the activity spectrum where LPA was classified as non-exercise activity thermogenesis (NEAT) [44]. Five studies examined changes in MVPA [58, 65], moderate-intensity physical activity (MPA), and/or vigorous-intensity physical activity (VPA) only [40, 41, 63], while another assessed both MVPA and energy expenditure [38]. Other outcome variables included energy expenditure (e.g. activity energy expenditure) [43, 45, 48] and time use variables (e.g. sitting time) [59, 61, 62, 66]. The analytical approach for studies that reported no evidence of compensation included 11 within-person analyses [20, 36, 41, 42, 48, 49, 51, 58, 61, 63, 68] and 12 between-group analyses [38, 39, 43, 45, 47, 53–55, 59, 60, 62, 66], whilst six used both analytical approaches [34, 35, 40, 44, 65, 70]. One study with mixed results (adolescent population) [33], reported that no compensation occurred in the location-based MVPA or overall MVPA component of their data (see Table 2).

### Behavioural studies (categories 2, 3 & 4)

Thirteen studies measured specific behaviours [62, 64–72], perceptions of compensation [63, 74], and/or mechanisms of compensation [73, 74] (see Table 3). Five studies were conducted with youth populations [63–65, 71, 72] and eight with adults [62, 66–70, 73, 74]. Ten quantitative studies contained a behavioural component recorded via a survey [63, 65–69, 71, 73] or activity diary [62, 64]. Two studies examined perceptions of compensation [63, 74], and two assessed potential mechanisms of compensation [73, 74].

**Table 3:** Potential behaviours and mechanisms of compensation

Author(s)/Study date	Behaviour method and type	Number of behaviours or topics	Behaviours Assessed/Reported Mechanisms	Included in quantitative comp analysis	Comp reported in behaviours
Clemes et al. [62]	Activity diary	5	Sitting in transport, sitting at work, sitting after work, total sitting time on workdays/non-workdays	Yes	No
Costigan et al. [63]	Survey, perceptions of compensation	1	Perceptions of compensation following activity intervention- 12.9% agreed that they were tired and did not want to participate in PE following a HIIT session, 13% agreed that participating in HIIT sessions made them less active in school breaks, and 19.4% agreed that participating in HIIT made them less active after school	N/A	No
Fremeaux et al. [64]	Activity diary	1	Daily activities (type, duration and time)	No	Yes
Gray et al. [74]	Qualitative interviews	9	Mechanisms of compensation (fatigue, drive to be inactive, time, fear of overexertion, motivation), implications (detracts from health benefits, does not detract from health benefits) and awareness of compensation (aware/unaware)	N/A	Yes
Goodman et al. [65]	Survey; cross checked with accelerometer	26	MVPA in own home, friend’s home, other home, school lessons, PE/games, clubs and tuition, non-home events, passive travel, school active travel, non-school active travel, structured sport, out of home play, other)	Yes	No
Jans et al. [66]	Survey	5	Total sitting time, sitting time at work, sitting travel to and from work, sitting housework	No	No
Matthews et al. [67]	Survey	17	Time spent sedentary and active time during personal care, leisure, work, transportation, shop/errands, other	Yes	No
McCormack et al. [68]	Survey	3	Transport walking, recreation walking	Yes	No
Nooijen et al. [69]	Survey/mechanism of compensation	2	Leisure time exercise, occupational PA	N/A	Yes
Radtke et al. [73]	Survey/mechanisms of compensation	1	Stair use and sedentary time	N/A	Yes
Siddique et al. [70]	Self-report; not described	1	Leisure time screen-time	No	No
Tanaka et al. [71]	Questionnaire	3	Time spent in each intensity while viewing TV and video, playing electronic games, and total screen time	Yes	Yes
Wilkin et al. [72]	Not clear	2	Transport to school, TV/video games	Yes	Yes

*Abbreviations*: *PE* Physical education, *HIIT* High intensity interval training, *MVPA* Moderate-to vigorous physical activity, *PA* Physical activity

#### Behaviours

The numbers of behaviours assessed ranged from 1 [64]-26 [65] and included passive and active travel [62, 66–68, 72], out-of-school activities [64, 65], leisure-time or personal activities [62, 64–67, 69, 70], occupational activity [62, 66, 67, 69], recreational walking [68], and screen time [70–72]. Behaviours were typically assessed using activity diaries and surveys, though one study combined a survey that was cross-checked with MVPA data collected using an accelerometer in settings [65] (see Table 3). In one study, it was unclear how behaviours were measured [72].

Of the 10 quantitative studies that included a behavioural component, four reported evidence of compensation [64, 69, 71, 72]. However, in three of these studies it was not clear whether compensation occurred in specific behaviours (i.e. data only reported the quantitative activity measures) [64, 71, 72]. In the remaining study, Nooijen and colleagues reported that adults who moved to a higher activity occupation compensated by decreasing their leisure-time exercise [69]. No evidence of compensation was reported in six studies (one in youth and five in adults) [62, 65–68, 70]. Based on time-use assessment, Jans et al. reported that those who had highly sedentary occupations did not compensate by decreasing leisure-time sedentary behaviour [66]. Further, Goodman et al. reported that there was no evidence of compensation in children aged 8–13 in any of the 26 MVPA behaviours assessed (e.g. MVPA in school lessons, P.E./games, active travel, etc. [65]) (see Table 3).

#### Mechanisms of compensation

Two studies examined potential mechanisms of compensation [73, 74]. In a sample of purposely selected participants who were identified as compensating their non-exercise physical activity during a 4-week structured activity intervention, reasons for activity compensation included fatigue, time constraints, lack of motivation, drive to be inactive (i.e. more activity means you can do less activity later), and fear of overexertion [74]. The second study, which examined the association between physical inactivity and compensatory health behaviours in young adults, reported that young adults with strong autonomous motivation believed that they could compensate their sedentary time by using the stairs later [73].

#### Perceptions of compensation

Two studies examined perceptions of compensation. Costigan and colleagues reported that compensation had not occurred when assessed using accelerometers, yet 13% of participants self-reported that their participation in the high intensity interval training (HIIT) sessions had made them less active during school breaks, and 19.4% thought they were less active after school [63]. In a qualitative study, Gray and colleagues reported that 56% of participants were unaware that they had compensated their activity [74].

### Quality assessment

The quality assessment for each study is shown in Table 4. The majority of studies (80%; *n* = 35) used device-based assessments, of which 24 studies included devices that were considered valid and reliable (54%). Examining activity across settings was evident in 72% of studies (*n* = 32). However, 86% of studies did not include an exposure (e.g. perturbation) as part of their design or did not deliver > 60% [29] of the exposure as intended (*n* = 13). Only two studies restricted activity during a time that would normally be active [36, 51], with one imposing activity during a time where children are normally inactive (i.e. timing of perturbation) [51]. Only 9 (20%) studies examined compensation across the activity spectrum [20, 21, 34, 35, 42, 45, 50, 51, 55].

**Table 4:** Modified McMaster for quality assessment of compensation studies

Authors	Selection Bias		Study design					Data Collection					Withdrawals/dropouts		Exposure		Analyses	
	Q1	Q2	Q3^**a**^	Q4	Q5^**a**^	Q6^**a**^	Q7^**a**^	Q8^**a**^	Q9a^**a**^	Q9b	Q9c	Q10^**a**^	Q11	Q12	Q13	Q14^**a**^	Q15^**a**^	Q16
Bagget et al. [20]	S	W	M	–	–	–	S	S	S	–	–	S	W	W	–	–	S	S
Carlson et al. [33]	S	W	W	–	–	–	S	S	S	S	–	W	–	W	–	–	S	S
Clemes et al. [62]	M	W	M	–	–	–	S	S	S	–	–	W	W	M	–	–	W	W
Clemes et al. [34]	M	W	M	–	–	–	S	S	S	–	–	S	W	S	–	–	S/W	W
Costigan et al. [63]	S	W	S	S	W	–	S	S	S	–	–	W	W	M	W	W	S	W
Cull et al. [35]	S	W	S	S	–	W	S	S	S	–	–	S	S	S	S	S	S/W	W
Dale et al. [36]	W	S	S	–	W	S	S	S	S	–	–	W	S	S	S	S	W	W
DiBlasio et al. [37]	W	W	S	W	W	–	S	S	W	–	–	W	S	S	W	W	S	S
Fremeaux et al. [64]	S	W	M	–	–	–	S	S	S	–	–	W	S	W	–	–	W	S
Gomersall et al. [38]	W	W	S	S	W	–	W	S	S	S	–	W	S	S	W	W	W	W
Goodman et al. [65]	M	W	M	–	–	–	S	S	S	W	–	W	W	W	–	–	S/W	W/S
Jakubec et al. [39]	S	S	M	–	–	–	S	S	S	–	–	W	S	M	–	–	W	W
Jans et al. [66]	S	W	W	–	–	–	S	W	W	–	–	W	–	–	–	–	W	W
Liguori et al. [40]	W	W	W	–	–	–	W	S	S	–	–	W	S	M	–	–	S	W
Long et al. [41]	S	W	W	–	–	–	S	S	S	–	–	W	–	W	–	–	S	S
Mackintosh et al. [42]	S	W	S	–	–	–	S	S	S	–	–	S	–	S	–	–	S	S
Massie et al. [43]	M	W	W	W	W	W	W	S	S	–	–	W	S	S	W	S	W	W
Matthews-Ewald et al. [44]	S	W	M	–	–	–	S	S	S	–	–	S	–	M	–	–	S/W	S
Matthews et al. [67]	S	W	W	–	–	–	S	W	W	–	–	S	–	–	–	–	S	S
McCormack et al. [68]	S	W	W	–	–	–	S	W	S	–	–	W	S	S	–	–	S	S
McLaughlin et al. [45]	W	W	S	W	W	–	W	S	W	W	–	S	W	W	W	W	S	W
Meijer et al. [46]	W	W	S	W	W	–	S	S	W	–	–	W	W	W	S	W	S/W	W
Morgan et al. [47]	S	W	M	–	–	–	W	S	S	–	–	W	W	W	–	–	W	W
Nooijen et al. [69]	S	W	M	–	–	–	S	W	S	–	–	W	S	W	–	–	W	S
O’Sullivan et al. [48]	W	W	S	W	W	W	S	S	W	–	–	W	S	S	S	W	S	W
Penning et al. [49]	W	W	S	S	W	–	S	S	S	–	–	W	S	M	S	W	S	W
Radtke et al. [73]	S	W	W	–	–	–	–	W	–	–	–	–	S	M	–	–	–	–
Ridgers et al. [50]	S	W	W	–	–	–	W	S	S	S	–	S	–	S	–	–	W	S
Ridgers et al. [51]	M	W	S	S	S	S	S	S	S	S	W	S	W	S	S	S	S	S
Ridgers et al. [52]	M	W	M	–	–	–	S	S	S	–	–	W	W	W	–	–	S	S
Ridgers et al. [21]	M	W	M	–	–	–	S	S	S	–	–	S	S	M	–	–	S	S
Rocha et al. [54]	W	W	S	S	W	W	S	S	W	W	–	W	S	S	W	W	W	S
Rocha et al. [53]	W	W	S	S	W	W	S	S	W	S	–	W	W	W	W	W	W	W
Saunders et al. [55]	W	W	S	S	W	W	S	S	–	–	–	S	W	W	W	W	W	S
Schubert et al. [56]	M	W	W	–	–	W	W	S	W	–	–	W	–	S	–	–	S	W
Schutz et al. [57]	S	W	S	S	–	W	W	S	S	–	–	W	W	W	W	M	W	W
Siddique et al. [70]	W	W	S	S	W	W	S	S	S	–	–	W	S	S	S	W	S	S
Stylianou et al. [58]	W	W	S	W	W	–	W	S	S	–	–	W	S	S	S	W	S	S
Tanaka et al. [71]	S	W	M	–	–	–	S	S	W	W	–	W	S	M	S	S	S	S
Tigbe et al. [59]	M	W	W	–	–	–	S	S	S	–	–	W	–	S	–	–	W	W
Turner et al. [60]	W	W	S	S	W	–	S	S	W	–	–	W	S	W	W	W	W	W
Vandelanotte et al. [61]	M	W	W	–	–	–	S	W	S	W	–	W	–	–	–	–	W	S
Wilkin et al. [72]	S	W	M/W	–	–	–	S	S	M	–	–	W	W	G1: S G2: M G3: W	–	–	W	S

Q1: Are the individuals recruited to participate likely to be representative of the intended target population? Is the analytical sample representative of the intended target population?

Q2: What percentage of selected individuals agreed to participate?

Q3: Indicate the study design

Q4: Was the study randomised?

Q5: Does the imposed activity occur at a time where the child is already active?

Q6: Does the restricted activity replace time that would normally be active?

Q7: Does the study examine activity across environments?

Q8: Is the activity measurement tool objective?

Q9: Is the measure valid and reliable?

Q10: Does the study examine activity across the whole activity spectrum?

Q11: Were individuals and dropouts reported in terms of numbers and/or reasons per group?

Q12: Indicate the percentage of participants completing the study/providing complete data

Q13: What percentage of participants received the allocated intervention or exposure of interest?

Q14: Was the full exposure delivered as intended?

Q15: Indicate the unit of analysis

Q16: Did they control for confounders?

Modified from the McMaster tool for quality assessment [29]. Full details regarding McMaster scoring can be found in Supplementary Information 3

*Abbreviations*: *S* Strong, *M* Moderate, *W* Weak, − Not applicable

^a^Compensation-specific criteria

## Discussion

This systematic review aimed to synthesise research that has investigated activity compensation in youth and adults and identify reasons for and/or awareness of compensatory changes that may have occurred. In general, this review did not find clear evidence that activity compensation occurs in either youth or adults. This may be due to the diverse approaches used to assess activity compensation, including different timeframes and study designs. However, 91% of the studies that reported evidence of compensation (*n* = 11), included assessing compensation as a primary (*n* = 9) or secondary (*n* = 1) aim, suggesting that purpose-designed studies are required to examine compensatory responses. Few studies examined perceptions and mechanisms of compensation, however; the results also suggested that while compensatory changes may occur, there was a lack of awareness of such responses in youth and adults.

This review builds on a previous review [24] through the inclusion of observational, experimental and intervention study designs. Interestingly, regardless of the study design utilised, no clear evidence of compensatory responses were observed, similar to a previous review, where mixed evidence of compensation was reported in children and adults [24]. It is worth noting that whilst 29 studies reported no evidence of compensation, 21% (*n* = 6) [35, 43, 53–55, 59] included a dietary compensation component, of which 67% (*n* = 4) [35, 53, 54, 59] reported some level of dietary compensation. As such, it could be that compensatory responses occur through the energy intake rather than energy expenditure. Further research is needed to examine the potential relationship between dietary and activity compensation. Another potential reason for the inconsistent results could be due to the way that compensatory changes were analysed. A range of analytical approaches were used by included studies to examine whether compensation occurs, including within-person and/or between person/group analyses. At least one-quarter of the observational studies [39, 47, 53, 54, 59, 62, 64, 66, 69, 72], interventions [38, 43, 60] and experimental studies [45, 55] only utilised between-person/group analyses, despite the activitystat hypothesis being a within-person hypothesis [19]. As such, this may impact the interpretation of findings. Studies should consider a within-person rather than between-group analytic approach to assess activity compensation given this is an individual response [19, 30]. Interestingly, of the studies that used between-group analyses, 25% reported evidence of compensation, whilst 35% of studies using within-person analyses reported evidence of compensation, indicating that when a purpose-driven methodological design is utilised, higher evidence of compensation is reported.

The time frame within which compensation would be expected to occur has been debated, with some suggesting that compensation would be unlikely to occur within-days [24], whilst others reporting that within-day compensatory changes were observed [52]. In this review, there was no clear evidence of a compensation time frame. Some studies reported evidence of compensation within [71] and/or between-days [52, 57], whilst others reported that compensation was evident over a longer period of time, such as between-seasons [37, 64, 69]. In contrast, some studies found no compensation within-day [36] and/or between-days [20, 42] or over longer periods of time [38, 43]. For intervention and experimental studies, when analysing two time points for compensation the days should be ‘comparable’ (i.e. structured similarly) to determine whether the changes observed may be attributed to compensatory responses [75] or variations driven by other factors (e.g. timetabling). However, few included studies reported considering the temporal nature of activity data in this way, and of those that did, only short time frames (e.g. < 24 h) were examined [51]. A previous review [24] suggested that compensation duration was synonymous with intervention duration, ranging from within-day to 4 years. However, it is unclear whether this reflects maintenance or changes in activity behaviours rather than compensation, as from a biological perspective, homeostatic processes could be expected to occur acutely. Future research assessing the time frame of compensation should initially examine acute responses before assessing changes over longer time periods.

The study design and compensation timeframe period are important when considering the perturbation of activity. Whilst a few studies examined the effect of a stimulus on participants’ activity [49, 55], the dose was not always reported. Few studies reported whether the stimulus occurred during a time when children were already active (e.g. during recess), making it difficult to determine whether the stimulus is eliciting a compensatory response or displacing usual activity [22, 30]. Only two studies restricted activity during normally active times (e.g. recess) [36, 51], despite compensatory responses being hypothesised to occur under such conditions [30]. A third study, which imposed sedentary time on children for an 8-h period, will have imposed inactivity on active periods of a child’s day. However, the amount of usual activity that was restricted during the imposed 8-h sedentary time period was not reported [55]. Lastly, 55% of the included quantitative studies were observational. Whilst observational studies may provide insights into intra-individual variability, the type and dose of perturbation were not described. As such, it is difficult to determine whether the dose of imposed activity and/or inactivity was outside the normal day-to-day variability (i.e. habitual activity patterns), to illicit a compensatory response [30]. In addition, it limits conclusions that any behaviour compensation was purely a biological response, or conversely a response influenced by the environments in which a person lives (e.g. structure of the day) [72]. Overall, future research should aim to report intra-individual variability to determine whether the perturbation exceeds such variability [76], and report the duration and activity intensity of the perturbation during the day.

Few studies (26%) considered changes in activity across the whole activity spectrum [20, 21, 34, 35, 42, 44, 45, 50, 51, 55, 67], despite the co-dependency of activity intensities occurring within a finite period (e.g. 24 h) [77]. The main activity intensity examined in both youth and adult populations was MVPA, which enables the assessment of changes in this intensity only. Arguably, responses to perturbation across activity intensities would be expected to occur across the whole activity spectrum, as all intensities would contribute to a daily set-point [21]. Given MVPA only constitutes 5% of a child’s waking hours [5] and 3% of an adult’s total day [78], if compensation were to occur, it is very likely to occur in lower intensities of the activity spectrum (LPA and SED) and not just in the intensity measured. Furthermore, it is possible that the findings generalised to other/daily behaviours, nor other population sub-groups. For example, some studies examined specific population groups (e.g. army cadets [40], office workers [66]), and outcomes reported were specific to those target groups (e.g. impact on MVPA, sitting time, etc.). Such findings are therefore specific to that population group and behaviour/intensity. Future studies should focus on assessment of compensation across the entire activity spectrum, and use statistical analyses that appropriately deal with co-dependency between these behaviours, such as compositional data analysis [77], to explore whether compensations may occur across the activity spectrum rather than within a single intensity. Further, future studies could consider sub-group analyses to see how compensation may occur across population groups.

Given the mixed findings and variability in methods and approaches it is difficult to draw conclusions concerning the existence of an activitystat and whether compensation occurs. While the one study [51] that scored ‘moderate’ or ‘strong’ across all compensation specific criteria of the quality assessment reported that compensation had occurred, this study was limited as participants did not participate in all three experimental conditions (imposed moderate- to vigorous physical activity, imposed light physical activity, and restricted physical activity) that is arguably needed to fully test the activitystat hypothesis. As some findings did report compensatory changes, this indicated that such responses do need to be considered in intervention designs moving forward. While compensation may not necessarily be harmful, it may depend on the response to a perturbation. Past literature has suggested that a new equilibrium around activity would indicate that individuals were able to modulate physical activity upwards and subsequently adjust the setpoint for physical activity [22, 72]. However, the issue therein, is that once a perturbation has been removed, there is little evidence to suggest that the modulated physical activity continues at that higher level [22]. These questions are important, yet complex to answer, without a clear understanding of whether compensation occurs (or not). As such, experimental studies are needed to determine what the impact of compensation is on health and whether different types of compensation have different health effects.

This systematic review was the first to examine mechanisms of or potential reasons for compensatory responses. Understanding how compensation may manifest behaviourally may enable researchers to specifically target behaviours at risk of compensatory changes. Ten studies examined potential compensatory changes in ~ 35 behaviours, yet few behaviours were consistently studied or clearly included in the compensatory analysis. Indeed, studies used different methods, such as temporal associations [65] and time use [66, 67], and MVPA in-school/out-of-school [65] and in different locations [64]. The one study that focused on a specific behaviour reported that adults who moved to a higher activity occupation compensated by decreasing their leisure-time exercise [69]. However, while two within-day measurements were analysed, the measurement time points were 4 years apart, making it difficult to understand whether this is truly a compensatory response, or if other factors (e.g., the environment) may also explain the results [69]. Overall, it is challenging to understand whether compensatory changes to behaviours occur, and if they do occur, how these may manifest between (e.g., walking to school, then public transportation home) or within behaviours (e.g., less active during a sports session). Future research should consider the use of purpose-designed surveys to examine time-use in different behaviours across settings, in conjunction with device-based assessments measurements.

Few studies examined potential mechanisms or reasons for compensatory behaviours. Fatigue, time constraints, lack of motivation, drive to be inactive, fear of overexertion, and perceived effort were identified as potential reasons or mechanisms of compensation in older adults [74]. Similarly, perceived effort to compensate combined with a drive to be inactive seemed prevalent in a study in young adults who reported that SED time could be compensated by a healthy behaviour such as taking the stairs [73]. To date, no studies have examined potential mechanisms (e.g., behavioural, psychological, or physiological mechanisms) of compensation in children. Despite this, results indicate that compensation may manifest in different ways within different population groups. Whilst qualitative research, for example, cannot determine whether compensatory changes occurred, it provides unique insights into potential mechanisms that could then be targeted by future interventions that aim to minimise such responses.

Lastly, few studies examined perceptions or awareness of any potential compensatory responses. In the qualitative study by Gray et al. [74], over half (56%) of participants (older adults) were unaware that they had compensated. Only one study measured self-reported perceived compensation [63]. Whilst most adolescent participants did not believe they compensated their activity because of the HIIT sessions, some thought they did compensate during (13%) or after school (19%) [63]. However, no further analyses were performed to see if their subjective experience matched the objective measurements or what traits, if any, these participants shared. It is unknown whether those that thought they compensated their activity actually did so, though it appears that, to some degree, people are aware that compensation may occur after activity. Future research should assess perceptions of activity compensation and examine differences across age groups (for example) and behaviour intensities. Understanding individual awareness of compensation, and any potential reasons for it, may identify why past activity interventions have had limited effectiveness, and inform the development of targeted interventions in the future.

### Strengths and limitations

This systematic review was the first to consider potential reasons for any compensatory changes observed. This review included all study designs, as well as behavioural studies, and was able to highlight a number of gaps in activitystat/activity compensation research. However, a few limitations must be acknowledged. Whilst the inclusion criteria were broad to reflect the way in which compensation has been examined to date, it was difficult to compare studies given the diverse range of approaches used and lack of standardised approaches (e.g., different statistical methods [within/between subjects], study designs [experimental, observational], etc.). This review aimed to synthesise all available activity compensation research; however, it was unable to draw firm conclusions as to the existence of activity compensation, and how it may manifest, given the variability in the methodology of studies that have examined this research area.

## Conclusion

Overall, this review found that compensation was observed in approximately one-third (32%) of youth and one-quarter (23%) of adult studies that utilised quantitative methods to examine the activitystat hypothesis. There was some evidence of compensation reported in studies where behaviours were assessed. However, there was substantial variability in study designs, time frames assessed, analytical approaches used, and behaviours examined in both the youth and adult studies, making it difficult to draw firm conclusions to the existence of the activitystat. Future research should consider focusing on experimental designs (with the type, timing and dose of perturbation reported), examining the whole activity spectrum, utilising a within-person analysis design across short and acute timeframes to assess whether compensation responses have occurred. Additionally, potential mechanisms of compensatory changes, and whether participants are aware of their compensation, should be assessed. This would provide valuable insights into what behaviours may be targeted in future interventions to negate compensatory changes.

## Supplementary Information

**Additional file 1.** PRISMA 2020 Checklist.

**Additional file 2.** Search strategy.

**Additional file 3.** Modified McMaster Quality Assessment Tool for Quantitative Activity Compensation Studies.

**Additional file 4.** Critical Review Form – Qualitative Studies (Version 2.0).

## Data Availability

The data supporting the conclusions of this article are included within Tables 1, 2, 3 and 4, and in its additional files (Supplementary Material Information S1, S2, S3, and S4).

## Abbreviations

MVPA: Moderate- to vigorous-intensity physical activity
SED: Sedentary behaviour
LPA: Light physical activity
PRISMA: Preferred Reporting Items for Systematic Reviews and Meta-Analyses
PAQ: Physical Activity Questionnaire
NEAT: Non-exercise activity thermogenesis
MPA: Moderate-intensity physical activity
VPA: Vigorous-intensity physical activity
HIIT: High intensity interval training
