# Supplementary material for: Exploring activity compensation amongst youth and adults: a systematic review
Source: Int J Behav Nutr Phys Act. 2022 Mar 12;19:25. doi: 10.1186/s12966-022-01264-6 (PMC8917655; doi:10.1186/s12966-022-01264-6)
Supplement: Supplementary file 2 — Additional file 2. Search strategy. [file 12966_2022_1264_MOESM2_ESM.docx]

**Exploring activity compensation amongst youth and adults: A systematic review**

Sports Medicine

Brittany A. Swelam^1^, Simone J.J.M. Verswijveren^1^, Jo Salmon^1^, Lauren Arundell^1^, & Nicola D. Ridgers^1^

^1^Deakin University, Geelong, Australia, Institute for Physical Activity and Nutrition, School of Exercise and Nutrition Sciences

Address for correspondence

Brittany Swelam

Institute for Physical Activity and Nutrition,

School of Exercise and Nutrition Sciences,

Deakin University,

221 Burwood Highway,

Burwood,

VIC 3125,

Australia

Email: [bswelam@deakin.edu.au](mailto:bswelam@deakin.edu.au)

**Supplementary Information 2. Search strategy**

| **Database** | **Date (dd.mm.yy)** | **Search String (AB, TI, SU/KW)** | **Limiters** |
| --- | --- | --- | --- |
| **EBSCOhost** (via Academic Search Complete, CINAHL complete, Education Source, Health Source: Nursing/Academic Edition, PsycINFO, SPORTdiscus with Full Text) | 24.05.2021 | [( compensat* N5 ("physical* activ*" or exercise or "energy expenditure" or "energy balance" or "sedentar*" or "sitting") ) OR (activitystat or energystat or energy N3 displac* )] AND [( child* or youth or adolesc* or teen* ) OR adult*] | Scholarly (peer-reviewed journals), Published date: 1999-present, Language: English Exclude: Medline |
| **MEDLINE Complete** | 24.05.2021 | [( compensat* N5 ("physical* activ*" or exercise or "energy expenditure" or "energy balance" or "sedentar*" or "sitting") ) OR (activitystat or energystat or energy N3 displac* )] AND [( child* or youth or adolesc* ) OR adult*] | Published date: 1999-present, Language: English |
| **Global Health** | 24.05.2021 | [( compensat* N5 ("physical* activ*" or exercise or "energy expenditure" or "energy balance" or "sedentar*" or "sitting") ) OR (activitystat or energystat or energy N3 displac* )] AND [( child* or youth or adolesc* ) OR adult*] | Published date: 1999- present |
| **EMBASE** | 24.05.2021 | ((compensat* NEAR/5 ('physical* activ*' OR exercise OR 'energy expenditure' OR 'energy balance' or 'sedentar*' or 'sitting')):ab,ti,kw) AND [1999-2021]/py OR (activitystat OR energystat OR ((energy NEAR/3 displac*):ab,ti,kw)) AND [1999-2021]/py AND (child*:ab,ti,kw OR youth:ab,ti,kw OR adolesc*:ab,ti,kw OR teen*:ab,ti,kw) AND [1999-2021]/py OR adult*:ab,ti,kw AND [1999-2021]/py | Published date: 1999-present, Language: English, Sources: EMBASE |
| **Scopus** | 24.05.2021 | ( ( TITLE-ABS-KEY ( compensat*  W/5  ( "physical activity"  OR  exercis*  OR  "energy expenditure"  OR  "energy balance"  or "sedentar*" or "sitting") ) AND  PUBYEAR  >  1998 ) OR  ( TITLE-ABS-KEY ( activitystat  OR  energystat  OR  energy  W/3  displac* )  AND  PUBYEAR  >  1998 ) )  AND  ( ( TITLE-ABS-KEY ( child*  OR  youth  OR  adolesc* OR teen* )  AND  PUBYEAR  >  1998 )  OR  ( TITLE-ABS-KEY ( adult* )  AND  PUBYEAR  >  1998 ) ) | Published date: 1999- present |
| **Web of Science** | 24.05.2021 | [(TI= (compensat* NEAR/5 ("physical* activ*" OR exercise OR "energy expenditure" OR "energy balance" or "sedentar*" or "sitting")) OR TS= (compensat* NEAR/5 ("physical* activ*" OR exercise OR "energy expenditure" OR "energy balance" or "sedentar*" or "sitting")) OR AB= (compensat* NEAR/5 ("physical* activ*" OR exercise OR "energy expenditure" OR "energy balance" or "sedentar*" or "sitting"))) AND LANGUAGE: (English) OR (TI= (ActivityStat or EnergyStat or Energy NEAR/3 displac*) OR AB= (ActivityStat or EnergyStat or Energy NEAR/3 displac*) OR TS= (ActivityStat or EnergyStat or Energy NEAR/3 displac*)) AND LANGUAGE: (English)] AND [(TI= (child* or youth or adolesc* or teen*) OR AB= (child* or youth or adolesc* or teen*) OR TS= (child* or youth or adolesc* or teen*)) AND LANGUAGE: (English) OR (TI= adult* OR AB= adult* or TS= adult*) AND LANGUAGE: (English)] | Published date: 1999-present, Language: English |
