# Supplementary material for: Exploring activity compensation amongst youth and adults: a systematic review
Source: Int J Behav Nutr Phys Act. 2022 Mar 12;19:25. doi: 10.1186/s12966-022-01264-6 (PMC8917655; doi:10.1186/s12966-022-01264-6)
Supplement: Supplementary file 3 — Additional file 3. Modified McMaster Quality Assessment Tool for Quantitative Activity Compensation Studies. [file 12966_2022_1264_MOESM3_ESM.docx]

**Exploring activity compensation amongst youth and adults: A systematic review**

Sports Medicine

Brittany A. Swelam^1^, Simone J.J.M. Verswijveren^1^, Jo Salmon^1^, Lauren Arundell^1^, & Nicola D. Ridgers^1^

^1^Deakin University, Geelong, Australia, Institute for Physical Activity and Nutrition, School of Exercise and Nutrition Sciences

Address for correspondence

Brittany Swelam

Institute for Physical Activity and Nutrition,

School of Exercise and Nutrition Sciences,

Deakin University,

221 Burwood Highway,

Burwood,

VIC 3125,

Australia

Email: [bswelam@deakin.edu.au](mailto:bswelam@deakin.edu.au)

**Modified McMaster Quality Assessment Tool**

**for Quantitative Activity Compensation Studies**

***Component Ratings***

A. Selection Bias

- 1. Are the individuals recruited to participate in the study likely to be representative of the intended target population? Is the analytical sample representative of the intended target population? ^1^
     1. Yes to both **Strong**
     2. Yes to one **Moderate**
     3. Neither **Weak**
     4. Can’t tell **Weak**
  2. What percentage of selected individuals agreed to participate? ^1^
     1. 80-100% agreement **Strong**
     2. 60-79% agreement **Moderate**
     3. Less than 60% agreement **Weak**
     4. Can’t tell **Weak**

B. Study Design

- 1. Indicate the study design^1^
     1. Experimental- **Strong**
     2. Randomised controlled trial **Strong**
     3. Observational- longitudinal **Moderate**
     4. Cross-sectional- time series **Moderate**
     5. Cross-sectional- regular **Weak**
     6. Other _____________ **Weak**
     7. Can’t tell **Weak**
  2. Was the study randomised? ^1^
     1. Yes **Strong**^*^
     2. No **Weak**
     3. Not applicable

**NOTE**: Refers to study design, not participant

- 1. Does the imposed activity occur at a time where the child is already active? ^2^
     1. No **Strong**^*^
     2. Yes **Weak**
     3. Not reported **Weak**
     4. Not applicable
  2. Does the restricted activity replace time that would normally be active? ^2^
     1. Yes **Strong**^*^
     2. No/Not reported **Weak**
     3. Not applicable
  3. Does the study examine activity across environments? ^2^
     1. Yes **Strong**^*^
     2. No/Not reported **Weak**

**NOTE:** Across environments refers to varying locations (e.g. in school and outside of school)

C. Data Collection

- 1. Is the activity measurement tool objective? ^2^
     1. Yes **Strong**^*^

(Accelerometer, heart rate monitor, pedometer, direct observation)

- - 1. No **Weak**

(Self-report, proxy report, etc.)

**a.)** Is the measure valid and reliable? ^1^

- - 1. Yes to both **Strong**
    2. Yes to one **Moderate**
    3. No **Weak**
    4. Can’t tell **Weak**

**b.)** Is the measure valid and reliable? ^1^

1. Yes to both **Strong**
2. Yes to one **Moderate**
3. No **Weak**
4. Can’t tell **Weak**
5. Not applicable

**c.)** Is the measure valid and reliable? ^1^

1. Yes to both **Strong**
2. Yes to one **Moderate**
3. No **Weak**
4. Can’t tell **Weak**
5. Not applicable

**Q10.** Did they examine the whole activity spectrum (SED, LPA, MVPA)?*

1 Yes **Strong**

2 No **Weak**

D. Withdrawals and Drop-outs

**Q11.** Were individuals and drop outs reported in terms of numbers and/or reasons per group? ^1^

1. Yes **Strong**
2. No **Weak**
3. Can’t tell/Not reported **Weak**
4. Not applicable

**Q12.** Indicate the percentage of participants completing the study/providing complete data: ^1^

1. 80-100% **Strong**
2. 60-79% **Moderate**
3. Less than 60% **Weak**
4. Can’t tell/Not reported **Weak**

**NOTE**: participant compliance

E. Exposure integrity

**Q13.** What percentage of participants received the allocated intervention or exposure of interest? ^1^

1. 80-100% **Strong**
2. 60-79% **Moderate**
3. Less than 60% **Weak**
4. Can’t tell **Weak**
5. Not applicable

**NOTE**: those that did not receive intended intervention

**Q14.** Was the full exposure delivered as intended? ^1^

1. 80-100% **Strong**
2. 60-79% **Moderate**
3. Less than 60% **Weak**
4. Can’t tell **Weak**
5. Not applicable

F. Analyses

**Q15.** Indicate the unit of analysis ^2^

1. Within person **Strong**
2. Between group **Weak**
3. Can’t tell **Weak**

**Q16.** Did they control for confounders?

1. Yes **Strong**^*^
2. No **Weak**
3. Can’t tell/Not reported **Weak**
