# Supplementary material for: Exploring activity compensation amongst youth and adults: a systematic review
Source: Int J Behav Nutr Phys Act. 2022 Mar 12;19:25. doi: 10.1186/s12966-022-01264-6 (PMC8917655; doi:10.1186/s12966-022-01264-6)
Supplement: Supplementary file 4 — Additional file 4. Critical Review Form – Qualitative Studies (Version 2.0). [file 12966_2022_1264_MOESM4_ESM.pdf]

Critical Review Form - Qualitative Studies (Version 2.0)

© Letts, L., Wilkins, S., Law, M., Stewart, D., Bosch, J., & Westmorland, M., 2007
McMaster University

CITATION:

Gray et al. 2018

	Comments
**STUDY PURPOSE:**<br><br>Was the purpose and/or research question stated clearly?<br>☑ yes<br>○ no	Outline the purpose of the study and/or research question.<br><br>Thus, a qualitative investigation ofthe causes ofcompensation among older adults who reduced NEPA in response to structured PA is warranted, and this study aimed to address this research gap using qualitative methods to explore complex aspects of older adults' opinions regarding the mechanismsofPAcompensation in greater depth. To the best ofthe authors' knowledge, this study is the first to q ualitatively investigate PA compensation among older adults thereby providing a unique contribution to the literature.
**LITERATURE:**<br><br>Was relevant background literature reviewed?<br>☑ yes<br>○ no	Describe the justification of the need for this study. Was it clear and compelling?<br>The justification included a lack of clear evidence around the psychological physiological mechanisms that may occur with the activitystat hypothesis. The justification was clear and compelling
	How does the study apply to your practice and/or to your research question? Is it worth continuing this review?[1]<br><br>This applies as we are hoping to collate any evidence about how compensation may manifest, perceptions of compensation, and potential drivers of compensation
**STUDY DESIGN:**<br><br>What was the design?<br>○ phenomenology<br>○ ethnography<br>○ grounded theory<br>○ participatory action research<br>☑ other<br>restrospective qualitative process evaluation	Was the design appropriate for the study question? (i.e., rationale) Explain.<br>Yes; this was a retrospective qualitative process evaluation to determine potential compensatory mechanisms in participants who had compensated their activity in a previous activity intervention. Interviews are suitable to the study's objectives.

1 When doing critical reviews, there are strategic points in the process at which you may decide the research is not applicable to your practice and question. You may decide then that it is not worthwhile to continue with the review.

Was a theoretical perspective identified?<br>☑ yes<br>○ no	Describe the theoretical or philosophical perspective for this study e.g., researcher's perspective.<br>The activitystat hypothesis
Method(s) used:<br>○ participant observation<br>☑ interviews<br>○ document review<br>○ focus groups<br>○ other<br>______________________	Describe the method(s) used to answer the research question. Are the methods congruent with the philosophical underpinnings and purpose?<br>Post process qualitative interviews; these were suitable to explore indivudal compensatory resposes, given that compensation is an individual response and it is thought that mechanisms of and reasons for compensation may be different in individuals
SAMPLING:<br><br>Was the process of purposeful selection described?<br>☑ yes<br>○ no	Describe sampling methods used. Was the sampling method appropriate to the study purpose or research question?<br>Yes, the sample was selected from a group of known 'compensators' in a previous intervention.
Was sampling done until redundancy in data was reached?[2]<br>☑ yes<br>○ no<br>○ not addressed	Are the participants described in adequate detail? How is the sample applicable to your practice or research question? Is it worth continuing?<br>The particpants are described in detail that is suitable to the study.
Was informed consent obtained?<br>○ yes<br>○ no<br>☑ not addressed	
DATA COLLECTION:<br><br>Descriptive Clarity<br>Clear & complete description of<br>site: ☑ yes ○ no<br>participants: ☑ yes ○ no<br><br>Role of researcher & relationship with participants:<br>☑ yes ○ no<br><br>Identification of assumptions and biases of researcher:<br>○ yes ☑ no	Describe the context of the study. Was it sufficient for understanding of the "whole" picture?<br>Information about the previous intervention was missing, however a description of how the participants were deemed 'compensators' was included<br><br>What was missing and how does that influence your understanding of the research?

2 Throughout the form, "no" means the authors explicitly state reasons for not doing it; "not addressed" should be ticked if there is no mention of the issue.

**Procedural Rigour**<br>Procedural rigor was used in data collection strategies?<br>☑ yes<br>○ no<br>○ not addressed	Do the researchers provide adequate information about data collection procedures e.g., gaining access to the site, field notes, training data gatherers? Describe any flexibility in the design & data collection methods.<br>This study is described in detail, however there is no reference to the previous study where the participants were selected from.
**DATA ANALYSES:**<br><br>**Analytical Rigour**<br>Data analyses were inductive?<br>☑ yes ○ no ○ not addressed<br><br>Findings were consistent with & reflective of data?<br>○ yes ○ no N/A	Describe method(s) of data analysis. Were the methods appropriate? What were the findings?<br>It seems to be a hybrid of inductive and deductive. There was an ovrearching theme in mind, however themes were allowed to develop naturally within the overarching theme. There were several findings related to mechanisms of comensation, awarenss of compensation, and implications of compensation. This was the first study of it's kind so there is no previous data to compare it to.
**Auditability**<br>Decision trail developed?<br>☑ yes ○ no ○ not addressed<br><br>Process of analyzing the data was described adequately?<br>☑ yes ○ no ○ not addressed	Describe the decisions of the researcher re: transformation of data to codes/themes. Outline the rationale given for development of themes.<br><br>Methodology was described in line with Braun and Clark's thematic analysis recommendations. No rationale provided apart from supporting literature.
**Theoretical Connections**<br>Did a meaningful picture of the phenomenon under study emerge?<br>○ yes<br>☑ no	How were concepts under study clarified & refined, and relationships made clear? Describe any conceptual frameworks that emerged.<br>Conceptual frameworks not relevant in a traditional sense. Activitystat hypothesis/ activity compensation as a guiding principle.

OVERALL RIGOUR<br>Was there evidence of the four components of trustworthiness?<br>Credibility ☑ yes ○ no<br>Transferability ☑ yes ○ no<br>Dependability ☑ yes ○ no<br>Comfirmability ☑ yes ○ no	For each of the components of trustworthiness, identify what the researcher used to ensure each.<br>Provided background rationale and full explanations of process throughout, had experienced qualitative researchers performing the analysis, provided full interview schedule<br><br>What meaning and relevance does this study have for your practice or research question?<br>This is the first study that explored compensation from a qualitative perspective. It provided interesting exploratory/introductory insights and considerations into compensatory responses in older adults.
CONCLUSIONS & IMPLICATIONS<br><br>Conclusions were appropriate given the study findings?<br>☑ yes ○ no<br><br>The findings contributed to theory development & future OT practice/ research?<br>☑ yes ○ no	What did the study conclude? What were the implications of the findings for [redacted] (practice & research)? What were the main limitations in the study?<br>This was different from a typical qualitative interview study as it was a post trial retrospective process evaluation that aimed to explore compensation in those that had compensated their activity. It was the first of it 's kind and was able to provide considerations of how compensation may manifest and why, making it valuable for it's unique methodogy and novel findings
